# Hepatoprotective effects of leaf extract of *Annona senegalensis* against aflatoxin B_1_ toxicity in rats

**DOI:** 10.4102/ojvr.v91i1.2133

**Published:** 2024-03-11

**Authors:** Rhulani Makhuvele, Kenn Foubert, Nina Hermans, Luc Pieters, Luc Verschaeve, Esameldin Elgorashi

**Affiliations:** 1 Department of Toxicology and Ethnoveterinary Medicine, Faculty of Public Health and Zoonoses, Agricultural Research Council-Onderstepoort Veterinary Research, Onderstepoort, Pretoria, South Africa; 2 Department of Paraclinical Sciences, Faculty of Veterinary Sciences, University of Pretoria, Pretoria, South Africa; 3 Natural Products and Food Research and Analysis (NatuRA), Department of Pharmaceutical Sciences, Faculty of Pharmaceutical, Biomedical and Veterinary Sciences, University of Antwerp, Antwerp – Wilrijk, Belgium; 4 Department of Risk and Health Impact Assessment, Sciensano (formerly Scientific Institute of Public Health), Brussels, Belgium; 5 Department of Biomedical Sciences, Faculty of Pharmaceutical, Biomedical and Veterinary Sciences, University of Antwerp, Antwerp – Wilrijk, Belgium

**Keywords:** aflatoxin B_1_, toxicity, hepatoprotective activity, Sprague-Dawley rats, *Annona senegalensis*

## Abstract

Global aflatoxin contamination of agricultural commodities is of the most concern in food safety and quality. This study investigated the hepatoprotective effect of 80% methanolic leaf extract of *Annona senegalensis* against aflatoxin B_1_ (AFB_1_)-induced toxicity in rats. *A. senegalensis* has shown to inhibit genotoxicity of aflatoxin B_1_ *in vitro*. The rats were divided into six groups including untreated control, aflatoxin B_1_ only (negative control); curcumin (positive control; 10 mg/kg); and three groups receiving different doses (100 mg/kg, 200 mg/kg, and 300 mg/kg) of *A. senegalensis* extract. The rats received treatment (with the exception of untreated group) for 7 days prior to intoxication with aflatoxin B_1_. Serum levels of aspartate aminotransferase, alanine aminotransferase, alkaline phosphatase, lactate dehydrogenase, and creatinine were measured. Hepatic tissues were analysed for histological alterations. Administration of *A. senegalensis* extract demonstrated hepatoprotective effects against aflatoxin B_1_-induced toxicity *in vivo* by significantly reducing the level of serum aspartate aminotransferase and alanine aminotransferase and regenerating the hepatocytes. No significant changes were observed in the levels of alkaline phosphatase, lactate dehydrogenase, and creatinine for the AFB_1_ intoxicated group, curcumin+AFB_1_ and *Annona senegalensis* leaf extract (ASLE)+AFB_1_ (100 mg/kg, 200 mg/kg, and 300 mg/kg body weight [b.w.]) treated groups. *Annona senegalensis* is a good candidate for hepatoprotective agents and thus its use in traditional medicine may at least in part be justified.

**Contribution:**

The plant extract investigated in this study can be used in animal health to protect the organism from toxicity caused by mycotoxins.

## Introduction

Contamination of agricultural commodities with spoilage fungi and their mycotoxins is a prominent worldwide public health and food safety concerns (Fernandes et al. 2021). Food and feed are the most exposed agricultural commodities that are vulnerable to fungal contamination in different areas. This contamination also exposes humans and animals to different dietary mutagens on a daily basis (Medalcho et al. 2023). Some of these toxigenic fungi produce genotoxic and mutagenic secondary metabolites. Aflatoxin B_1_ (AFB_1_) is one of the most common dietary mutagenic, genotoxic, carcinogenic, immunosuppressive, and teratogenic metabolites produced by *Aspergillus flavus and Aspergillus parasiticus* (Medalcho et al. 2023; Zarev et al. 2020). It contaminates agricultural commodities, thus affecting food and feed quality as well as safety. Aflatoxin B_1_ is classified as a group 1 human carcinogen by the International Agency for Research on Cancer (IARC 2002).

Humans and animals are exposed to AFB_1_ through contact, ingestion, and inhalation. This exposure can lead to chronic and acute toxicity. Chronic exposure to AFB_1_ results in low to moderate AFB_1_ intoxication and this is linked to hepatocellular carcinogenesis. Acute AFB_1_ exposure by ingesting high doses of AFB_1_ causes hepatotoxicity (Benkerroum 2020). Aflatoxin B_1_ is metabolised in the liver by the cytochrome enzymes. The metabolic conversion of AFB_1_ generates a highly reactive AFB_1_ species known as AFB_1_-exo-epoxide and other reactive oxygen species such as hydroxyl radicals, superoxide anions, and hydrogen peroxide (Awuchi et al. 2021; Makhuvele et al. 2022). These reactive oxygen species can lead to DNA lesions, lipid peroxidation, and cell damage. Aflatoxin B_1_ toxicity differs among different species, with some organisms being more susceptible than others. The main mechanism of toxicity of AFB_1_ in both humans and animals is through the induction of oxidative stress (Lin et al. 2022).

Oxidative stress resulting from AFB_1_ can be equally prevented by the use of botanical extracts and synthetic antioxidants, but some synthetic antioxidants are toxic (Rasouli, Nayeri & Khodarahmi 2022). Botanicals have been used since the existence of humanity to treat various ailments and are part of a trillion dollar growing industry. The World Health Organization (WHO) has estimated that 80% of the world’s population still relies on herbal medicine to treat diseases with over 40% of pharmaceutical formulations derived from natural products (WHO 2018). Research has shown that botanical extracts possess protective agents that can act against toxicity of AFB_1_. Plants are known to possess compounds that can interact with reactive oxygen species (ROS) and protect the cell. They are rich in antioxidant, anticarcinogenic, and antimutagenic constituents, anti-inflammatory, hepatoprotective, and antigenotoxic constituents (Bansal & Chinmayee 2022; Makhuvele et al. 2020; Makhuvele et al. 2022). Curcumin is well-known for its chemoprotective and hepatoprotective properties against AFB_1_-induced hepatocarcinogenesis and hepatotoxicity in rodents, broilers, and ducklings (Dai et al. 2022; Mohajeri et al. 2018; Pauletto et al. 2020).

The Annonaceae family is among the most promising families against carcinogens (Emiliana et al. 2019; Nugraha et al. 2019; Yakubu et al. 2020). *Annona senegalensis* Persoon has been used in traditional medicine to treat microbial and parasitic infections and tumours in human and animals (Mulholland et al. 2000; Okhale et al. 2016). Previously, we reported the antigenotoxic potential of *A. senegalensis* against AFB_1_-induced genotoxicity in *in vitro* assays (Makhuvele et al. 2018). Furthermore, the antioxidant and hepatoprotective effect of stem bark extracts of *A. senegalensis* against carbon tetrachloride-induced damage was also reported (Omeke et al. 2019). *Annona senegalensis* has been reported to possess phytoconstituents including acetogenins, tannins, saponins, alkaloids, steroids, glycosides, anthocyanins, essential oils, and minerals, which have been reported to contribute to its anticancer activities (Al Kazman, Harnett & Hanrahan 2022; Okechukwu et al. 2023; Omeke et al. 2019). This study is aimed at investigating the hepatoprotective effects of hydromethanolic leaf extract of *A. senegalensis* against AFB_1_ toxicity on Sprague-Dawley rats using biochemical and histopathological parameters.

## Research methods and design

### Chemicals

AFB_1_ (≥ 98%) and curcumin (≥ 75%) were purchased from Sigma Aldrich. Propylene glycol (Univar grade) was bought from Merck, and methanol (HPLC grade) was obtained from VWR.

### Plant collection and identification

*Annona senegalensis* (Annonaceae) leaves were collected from Lowveld National Botanical Gardens in Mpumalanga province of South Africa in March 2015. Mr. Willem Froneman from the South African National Biodiversity Institute confirmed the identity of the plant material. A voucher specimen (PRU 122755) was deposited at the H.G.W.J. Schweickerdt Herbarium of the University of Pretoria.

### Plant processing

The leaf material of *A. senegalensis* was rinsed with distilled water to remove soil debris and dried in an oven set at 45°C for about 3 days. Thereafter, it was pulverised to a powder. Four hundred grams of the powdered plant material was exhaustedly extracted with 80% methanol by maceration at room temperature. Then, the plant extract was filtered through Whatman No.1 filter paper and concentrated to dryness using a rotary evaporator set at 40 ^o^C to give a residue of 120 g (yield 30%).

### Animal studies

The studies were carried out as described previously by Makhuvele et al. (2022).

#### Animals

Forty-eight healthy 7 week old Sprague-Dawley male rats (150 g – 200 g) were purchased from South African Vaccine Producers (SAVP) in Johannesburg, South Africa. The rats were caged in pairs with enrichment items such as wooden sticks for gnawing, tissue paper, and egg containers under a controlled temperature of ±22 ^o^C, and humidity at ±50% in a light and dark cycle of 12 h. They were fed with a conventional rodent diet and water, available *ad libitum* for the duration of the study. The rats were allowed to acclimatise and were carefully monitored under laboratory conditions for 5 days prior to treatment. They were maintained as per the University of Pretoria Animal Ethics Committee with the protocol (V073-15) guidelines.

#### Hepatoprotective study

The rats were randomly grouped into six groups (*n* = 8). Group 1: did not receive any treatment (untreated control). Group 2: received 25% propylene glycol (negative control). Group 3: animals treated with 10 mg/kg body weight (b.w.) curcumin (positive control). The concentration of curcumin was selected based on the findings from previous studies where the dosage produced a marked antioxidant and potent hepatoprotective effect against aflatoxin B_1_ in other models of induced liver injury (Soni, Rajan & Kuttan 1992). The dose of *Annona senegalensis* leaf extract (ASLE) was selected based on previous studies conducted by Rotimi et al. (2017); Sathya, Kokilavani & Ananta Teepa (2012), while the dose of AFB_1_ was chosen based on studies by Rotimi et al. (2017); Zarev et al. (2020). Group 4–6 received 100, 200, and 300 mg/kg b.w. ASLE, respectively. All treatments were administered by oral gavage once a day for 7 consecutive days. On day 8 of the study, all rats (except the untreated control group) were treated with AFB_1_ (1 mg/kg b.w.) by oral gavage. After 72 h, the rats were euthanised with isoflurane, the livers were removed, and then fixed in 10% buffered formalin for histological analysis.

#### Determination of serum parameters

The protective effect of the ASLE was assessed by measuring the levels of the following serum enzymes: alanine aminotransferase (ALT), aspartate aminotransferase (AST), alkaline phosphatase (ALP), lactate dehydrogenase (LDH), and creatinine. Blood samples were collected from the lateral tail vein of each rat on day 0 and by cardiac puncture on day 10 following anaesthesia with isoflurane and prior to sacrifice. The blood samples were sent to Clinpath diagnostic laboratory for evaluation of serum enzymes. The analysis was performed following Clinpath standard operating procedures using COBAS INTEGRA 400 kits plus automatic Chemistry Analyzer (Roche Diagnostics; Mannheim, Germany).

#### Histopathological studies

After euthanasia of the animals, the liver tissue samples were analysed for histopathological changes following standard methods for histopathological examination.

### Statistical analysis

Analysis of variance (ANOVA) between the groups was performed using SAS version 9.3 (TS1M2). The standardised residuals were tested for deviations from normality using Shapiro–Wilk’s test. Data are presented as mean ± standard deviation. The data were considered significant at *p* ≤ 0.05.

### Ethical considerations

Ethical clearance to conduct this study was obtained from the University of Pretoria Animal Ethics Committee (project no. V073-15).

## Results

### Effect of treatment on animal well-being

All rats survived throughout the experimental period. No clinical signs of illness or abnormalities were reported in all rats treated with ASLE, or in the negative and positive control groups. The chosen dosages had no major effects on the physiology of the rats.

### Effect of ASLE on aflatoxin B_1_-induced toxicity on selected serum enzymes

**FIGURE 1:**
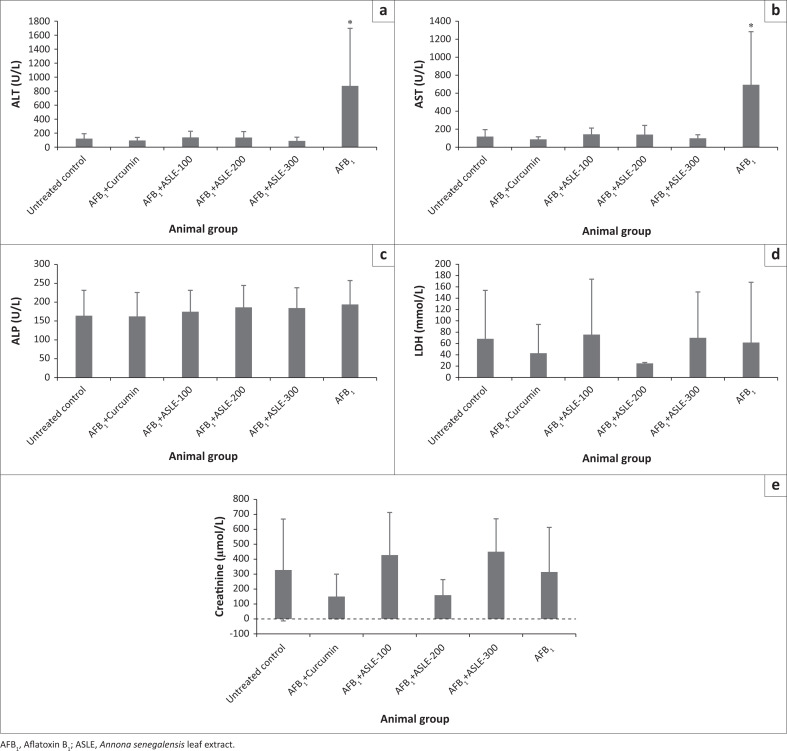
Effect of aflatoxin B_1_ on (a) alanine aminotransferase; (b) aspartate aminotransferase; (c) alkaline phosphatase; (d) lactate dehydrogenase and (e) creatinine levels of the rats treated with plant extracts and control substances. Data were expressed as mean ± s.d. (*n* = 8). Significant difference at *p* ≤ 0.05 is indicated by (*).

**FIGURE 2:**
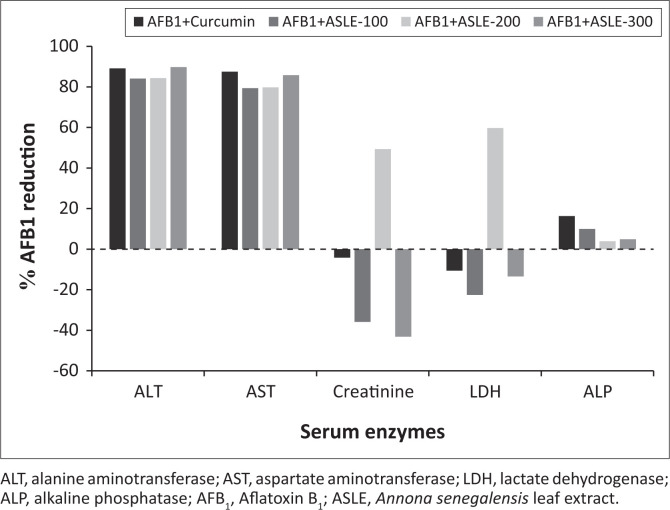
Percentage reduction of serum enzymes (alanine aminotransferase, aspartate aminotransferase, alkaline phosphatase, lactate dehydrogenase and creatinine) by curcumin and ASLE (100 mg/kg – 300 mg/kg) in relation to the AFB_1_ group.

The effects of AFB_1_ intoxication as well as the hepatoprotective effect of ASLE and curcumin on serum biochemical parameters are presented in Figure 1a–e. A significant increase in the levels of ALT and AST was observed in the rats treated with AFB_1_ only (*p* < 0.001) when compared with ASLE+AFB_1_ (100 mg/kg, 200 mg/kg and 300 mg/kg b.w.) and curcumin + AFB_1_ groups, which exhibited a significant decrease in the levels of AST and ALT (*p* < 0.001). There was a tendency to a dose-dependent response to the plant extract ASLE, although no statistical difference was observed between the tested doses. Curcumin and ASLE (100 mg/kg b.w. – 300 mg/kg b.w.) reduced AFB_1_-induced increase of serum ALT and AST by 84% – 90% and 79% – 86%, respectively (Figure 2).

Intoxication with AFB_1_ alone had no significant effect on the levels of ALP, LDH, and creatinine in the blood. No significant changes were observed in the levels of ALP (0.876), LDH (*p* = 0.800), and creatinine (*p* = 0.079) for the untreated group, AFB_1_ intoxicated group, curcumin + AFB_1_ and ASLE + AFB_1_ (100 mg/kg, 200 mg/kg and 300 mg/kg b.w.) treated groups. The relative AFB_1_ reduction of ALP was low, ranging between 4% and 16%, whereas the relative AFB_1_ reduction of creatinine and LDH was very low and similar to the control (Figure 2). This implies that AFB_1_ did not have any effect on the levels of ALP, LDH, and creatinine in all treatment groups.

### Histopathological analysis

**FIGURE 3:**
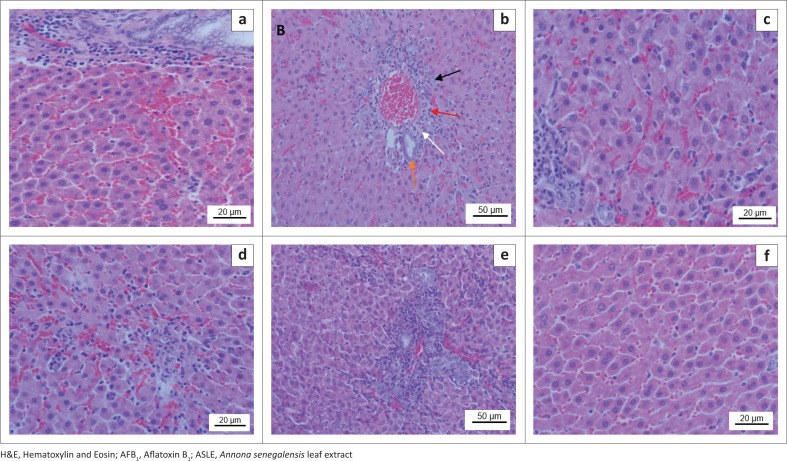
Photomicrograph of the liver cross section of a Sprague-Dawley rat (H&E, × 40). (a) Untreated control group; (b) AFB_1_-induced toxicity; slight to mild hepatocellular injury, bile duct proliferation (black arrow), lymphoplasmacytic infiltrate (red arrow), fibrosis (white arrow), normal bile duct (orange arrow). (c) AFB_1_+ curcumin; (d) AFB_1_+ ASLE 100 mg/kg; (e) AFB_1_+ ASLE 200 mg/kg; (f) AFB_1_+ ASLE 300 mg/kg showing the hepatoprotective effect of the ASLE and curcumin.

The histopathological analysis showed mild to moderate changes in the hepatic architecture of rats intoxicated with AFB_1_. The predominant hepatic lesions include hepatocellular injury with bile duct proliferation and hyperplasia, hydropic degeneration, necrosis, fibrosis, and portal lymphoplasmacytic infiltrates (Figure 3).

## Discussion

This study investigated the protective effects of the hydromethanolic extract of *A. senegalensis* leaves against AFB_1_ hepatotoxicity. The outcomes of this study indicated that AFB_1_ significantly elevated the levels of AST and ALT in rats treated with AFB_1_ only. This increase in the levels of AST and ALT in the AFB_1_ treated group is an indication of hepatic oxidative damage as these enzymes are released into the blood only when there is tissue or cellular damage (Yakubu et al. 2020). The higher levels and higher standard deviations observed in the negative control group were because of inter-individual differences in the metabolism, bioavailability, and absorption of AFB_1_ by the rats. All tested doses of ASLE and curcumin were capable of ameliorating the hepatotoxic effect of AFB_1_, by normalising the serum levels of AST and ALT in the blood. These serum enzymes are found in low amount during a normal physiological state, but they increase in response to tissue or cell damage (Omeke et al. 2019). However, the highest concentration of ASLE was effective in preventing AFB_1_-induced damages comparably with the low concentration. Aflatoxin B_1_ intoxication did not have any effect on the levels of ALP, LDH, and creatinine in all treated groups. These results revealed that AFB_1_ did not have any effect on muscle metabolism in rats, as the levels of creatinine waste remained the same before and after AFB_1_ treatment.

Histopathological examination of rat organs was performed to support the results from the biochemical analysis. Aflatoxin B_1_ treatment resulted in mild to severe hepatocellular injury characterised by bile duct proliferation, necrosis, fibrosis, hydropic changes, and lymphoplasmacytic infiltrate. These findings are in accordance with the results from previous studies (Makhuvele et al. 2022; Yaman, Yener & Celik 2016). Aflatoxin B_1_ causes oxidative stress, necrosis, cirrhosis, hepatocellular injury, and liver cancer, as it is the main organ for AFB_1_ metabolism as also reported by Guo et al. (2023). Minimal histopathological architectural damages of hepatic tissues and cells were observed in AFB_1_-intoxicated rats treated with different doses of ASLE and curcumin. However, these injuries were noticeably reduced to slight signs of sub-lethal non-specific hepatocellular injuries in rats treated with ASLE and curcumin, thus indicating the ameliorative effects of the plant extracts against AFB_1_-induced hepatotoxicity. The protective effects of curcumin are attributed to its antioxidant capacity, which includes scavenging of ROS. Its mechanism of action involves inhibiting of activation of AFB_1_ by phase I enzymes and activation of phase II enzymes through activation of the Nrf2 signalling pathway and its downstream genes (Das & Vinayak 2015; Li et al. 2019; Muhammad et al. 2018). These findings are in line with the study by Omeke et al. (2019), which demonstrated potent hepatoprotective activity of *A. senegalensis* against carbon tetrachloride-induced liver damage in rats. Furthermore, Yakubu and coworkers (2020) showed the protective ability of the *n*-hexane extract of *A. senegelensis* against diethylnitrosamine-induced hepatocellular carcinoma. Previous studies in our laboratory reported the *in vitro* antigenotoxic effect of *A. senegalensis* against AFB_1_ (Makhuvele et al. 2018). The hepatoprotective effects of ASLE against AFB_1_-induced hepatoxicity may be because of the presence of phytoconstituents such as phenolic compounds including tannins, anthocyanins, found in *A. senegalensis*, which have been reported to contribute to its anticancer activities (Babalola et al. 2021; Yakubu et al. 2020).

## Conclusion

The results obtained from this study demonstrated that ASLE has protective effects against AFB_1_-induced hepatotoxicity by reducing the levels of the serum enzymes AST and ALT and stimulating the hepatic regeneration. This protection is attributed to the induction of antioxidant activities by the phenolic contents of ASLE. *Annona senegalensis* is well-known in herbal medicine because of its biological activities against various ailments. Furthermore, determination of the bioactive phytoconstituents responsible for these protective activities and their mechanism of action is crucial for advancement of new hepatoprotective drugs and supplements.
